# Activation of Eosinophils and Mast Cells in Functional Dyspepsia: an Ultrastructural Evaluation

**DOI:** 10.1038/s41598-018-23620-y

**Published:** 2018-03-29

**Authors:** Hanne Vanheel, Maria Vicario, Werend Boesmans, Tim Vanuytsel, Eloisa Salvo-Romero, Jan Tack, Ricard Farré

**Affiliations:** 1 Translational Research Center for Gastrointestinal Disorders (TARGID), KU Leuven, Leuven, Belgium; 2 Digestive Diseases Research Unit, Institut de Recerca Vall d’Hebron, Department of Gastroenterology, Hospital Universitari Vall d’Hebron, Universitat Autònoma de Barcelona, Barcelona, Spain; 3 Centro de Investigación Biomédica en Red de Enfermedades Hepáticas y Digestivas (CIBERehd), Barcelona, Spain; 4 Department of Pathology, GROW-School for Oncology and Developmental Biology, Maastricht University Medical Center, Maastricht, The Netherlands

## Abstract

We recently identified mucosal mast cell and eosinophil hyperplasia in association with a duodenal impaired barrier function in functional dyspepsia (FD). We aimed to further describe the implication of these immune cells by assessing their activation state at the ultrastructural level and by evaluating the association between impaired epithelial integrity and immune activation. Duodenal biopsies were obtained from 24 FD patients and 37 healthy controls. The ultrastructure of mast cells and eosinophils was analyzed by transmission electron microscopy. Transepithelial electrical resistance and paracellular permeability were measured to evaluate epithelial barrier function. The type of degranulation in eosinophils and mast cells was piecemeal. Eosinophils displayed higher degree of degranulation in FD patients than in controls (p < 0.0001). Quantification revealed a decreased granular density in eosinophils of FD patients (p < 0.0001). The degree of degranulation in mast cells was similar in both groups. However, a more heterogeneous profile was found in the FD group (p < 0.0001). No association between epithelial integrity and the number and activation state of mucosal eosinophils and mast cells was found. We demonstrated ultrastructural changes in degranulation state of eosinophils and mast cells, suggesting that eosinophil and mast cell activation play a role in the pathophysiology of FD.

## Introduction

Functional dyspepsia (FD), characterized by a diversity of symptoms localized in the epigastric region, is one of the most common functional gastrointestinal disorders. The terminology ‘functional’ is used when no organic, systemic or metabolic abnormality can be identified after a standard diagnostic work-up^1^. The disorder has a high prevalence in western countries and is responsible for reduced quality of life and considerable economic burden on society^2^. Over the years, several mechanisms have been proposed to underlie dyspeptic symptom generation, including psychological abnormalities and gastric sensorimotor dysfunction^3,4^. Research recently also focused on changes in duodenal function, as studies have demonstrated duodenal hypersensitivity to acid and lipids^4^. Additionally, the latest reports showed that inflammatory mechanisms in the duodenal mucosa could be involved in the pathophysiology of FD^4^.

The continuously increasing number of studies reporting low-grade duodenal inflammation in FD were mainly based on the histological detection of a slightly but significant increased number of mucosal immune cells, mainly eosinophils and mast cells^4^. Few studies have attempted to evaluate activation of these cells, and this has been carried out by non-specific methodology using immunohistochemistry^3,5^. A more precise assessment of activation based on ultrastructural changes observed using transmission electron microscopy (TEM) has been extensively performed in other diseases^6^, but only one study has used this approach in a pediatric FD population revealing a high frequency of activated eosinophils^7^.

In a previous study, we demonstrated increased duodenal permeability in patients with FD and found a correlation between certain cell-to-cell adhesion proteins and low-grade inflammation^8^. Although these data indicate that there is an association between impaired integrity and inflammatory activity, we do not know whether increased permeability is the cause or consequence of low-grade inflammation or even an unrelated epiphenomenon. Earlier research already showed that mast cells and eosinophils can play a role in impaired epithelial barrier function. It has been reported that major basic protein (MBP) released by eosinophils can disrupt the epithelial barrier by downregulating expression of occluding, one of the proteins involved in tight junction barrier function^9^. Other studies found that the activation of eosinophils leads to the release of corticotrophin-releasing hormone, provoking mast cell degranulation, which in turn induces barrier dysfunction^10,11^. Conversely, impaired barrier function can also result in low-grade inflammation, as studies in animal models have demonstrated attenuation of inflammation after prevention of elevated intestinal permeability^12^.

In order to identify the implication of mucosal immunity in the pathophysiology of FD, the primary aim of this study was to assess activation of mast cells and eosinophils at the ultrastructural level in FD patients. As a secondary endpoint, we wanted to investigate the relationship between impaired integrity and eosinophil and mast cell activation in FD patients.

## Material and Methods

### Participants

Patients meeting Rome III criteria for FD^13^ were prospectively recruited from the outpatient clinic of the Department of Gastroenterology at the University Hospitals Leuven, a tertiary care referral centre. All patients were referred for the evaluation of dyspeptic symptoms for which upper gastroscopy with duodenal biopsies was required and found to be normal. All patients were *Helicobacter pylori* negative and underwent careful history taking, clinical examination and routine biochemistry. Healthy volunteers, as the control group, were recruited by a mailing list after exclusion of gastrointestinal symptoms or a history of gastrointestinal disease. Exclusion criteria for all participants were: intake of NSAIDs, corticosteroids or other immunosuppressive drugs in the preceding 6 months; diabetes or celiac disease; first-degree family members with type I diabetes, celiac disease or inflammatory bowel disease. Written informed consent was obtained prior to inclusion in the study. The protocol was approved by the ethics committee of the University Hospitals of Leuven. All methods were performed in accordance with our institution guidelines and regulations.

### Duodenal biopsies

During upper gastroduodenoscopy, biopsy specimens were taken with a standard biopsy forceps (Radial Jaw3, outside diameter 2.2 mm; Boston Scientific, Natick, Massachusetts, USA) from the second part of the duodenum by an experienced endoscopist (JT). Two biopsy samples were fixed in formalin and embedded in paraffin for immune cell counting by immunohistochemistry procedures. Another two biopsy specimens were fixed with 2.5% (v/v) glutaraldehyde (Sigma-Aldrich, St. Louis, Missouri, USA) and 2% (v/v) paraformaldehyde (Sigma-Aldrich) in 100 mM phosphate buffer (pH 7.4) for ultrastructural evaluation by TEM. Three biopsy samples (only from FD patients) were put in ice-cold oxygenated Krebs-Ringer bicarbonate for Ussing chamber experiments.

#### Immunohistochemistry

Duodenal biopsy sections were deparaffinized using general procedures, followed by blocking with REAL Peroxidase Blocking (Dako, Glostrup, Denmark) and Protein Blocking Solution (Dako) as previously described^5^. Eosinophils and mast cells were stained by incubating sections at room temperature for 60 min in mouse anti-MBP (1:20; AbD Serotec, Kidlington, UK) or 30 min in mouse anti-mast cell tryptase (1:200; Dako), respectively. Next, sections were incubated with secondary horse anti-mouse biotinylated antibody (1:200; Vector Laboratories, Burlingame, California, USA) and diaminobenzidine was used as the chromogen, followed by counterstaining with Harris’s haematoxylin. In a blinded manner, images of at least seven representative non-overlapping fields were taken on a BX41 Olympus microscope using Cell^F software (Olympus, Aartselaar, Belgium) with a 20× objective. The area of the lamina propria was measured using ImageJ software (National Institutes of Health); positive cells were counted and the results were expressed as the number of cells per mm².

#### Transmission electron microscopy

After fixation during 2 h, duodenal biopsy samples were rinsed with 100 mM phosphate buffer. Tissues were then post-fixed in 1% (w/v) osmium tetroxide containing 0.8% (w/v) of potassium hexacyanoferrate (III) (Sigma-Aldrich) for 2 h and then washed with 100 mM phosphate buffer (all these steps at 4 °C). Samples were dehydrated through a graded acetone series, infiltrated in Epon’s resin and polymerized for 48 h at 60 °C. Ultrathin sections (70 nm) were mounted in copper grids, contrasted with standard uranyle acetate and lead citrate double-staining, and observed in a Jeol JEM-1400 TEM equipped with a Gatan Ultrascan ES1000 CCD camera (Jeol LTD, Tokyo, Japan). Examinations were performed independently by one experienced investigator (MV) in a blinded manner on a minimum of 30 sections per biopsy sample. The general structure of each tissue was evaluated, and only samples containing both intact epithelium and lamina propria (assuring good fixation) were evaluated. Mast cells and eosinophils were identified based on their specific morphology. The analysis included the evaluation of the presence, type and degree of degranulation in both cell types at x1,000-15,000 magnification. Piecemeal degranulation (loss of intragranular electro-density without signs of intergranular or granule-to-cell membrane fusion) was identified as a marker of cell activation^7^. Presence and absence of degranulation were quantified in samples from all subjects included in the study and data were expressed as the percentage of subjects with or without degranulated cells. In samples with activated cells, the degree of degranulation was established as high (decreased density in >50% granules/cell) or low, and data were expressed as the percentage of subjects with high or low degranulation. Quantitative analysis of the density of eosinophilic granules was also performed using ImageJ. Histogram equalization was performed to standardize all TEM images over the full dynamic range (8 bit). Regions of interest were drawn over individual granules to determine their pixel intensity profile and average intensity (arbitrary units). Besides degranulation, the complexity of granular content in mast cells was assessed based on the granular structure (scrolls, crystals, particles) and classified as homogeneous (similar profile in >50% granules/cells) or heterogeneous. All analyses were performed in a blinded manner.

#### Ussing chamber experiments

Duodenal biopsy specimens were mounted in modified 3 mL Ussing chambers (Mussler Scientific Instruments, Aachen, Germany) as described previously^8^. Transepithelial electrical resistance (TEER) was recorded every 30 min during 2 h; the average of all time points of the three biopsy samples was taken and results are presented as Ω.cm². Passage through the biopsy specimens was evaluated with the paracellular probe FITC-dx4 (MW = 4,000 Da, 1 mg/mL; Sigma-Aldrich, St. Louis, USA). FITC-dx4 was added to the mucosal compartment and serosal samples were collected every 30 min during 2 h, of which the fluorescence level was measured using a fluorescence reader (FLUOstar Omega; BMG Labtech, Ortenberg, Germany). The average of time point 60, 90 and 120 min of the biopsy samples was taken and results are presented as pmol.

### Statistical analysis

Data are presented as mean ± SEM or median (IQR) depending on data distribution, which was checked by the Kolmogorov-Smirnov test. Differences between groups were evaluated using two-tailed unpaired *t* tests or Mann-Whitney *U* tests for continuous variables. Categorical variables were compared using the Fisher’s exact test or the Chi-square test. Pearson’s *r* or Spearman’s *ρ* were used to determine correlations when appropriate. We reported which p values remained significant after Bonferroni correction for multiple testing. One-way ANOVA, followed by post-hoc testing (Tukey), was used to analyze the difference in mean intensity per granule between no, low and high eosinophilic degranulation. The pixel intensity profile was calculated per eosinophilic granule and grouped in bins of 15 arbitrary units for each individual. Two-way ANOVA with Bonferroni correction was used to evaluate the intensity distributions. Statistical comparisons were performed using the GraphPad Prism software and values were considered statistically different when p < 0.05.

## Results

### Study population

Twenty-four patients with FD fulfilling Rome III criteria^13^ (8 men, 16 women; age 30.5(22.8–42.5) years, BMI 20.7(19.7–22.5) kg m^−2^) and 37 healthy volunteers (18 men, 19 women; age 24.0(22.0–27.5) years, BMI 22.8(20.8–24.2) kg m^−2^) were included in the study. There was no significant difference in gender (p = 0.29) and age (p = 0.06) between both groups, but the BMI was lower in the FD patient group (p = 0.01).

### Patients with functional dyspepsia are characterized by an increased number of eosinophils and mast cells

**Figure 1:**
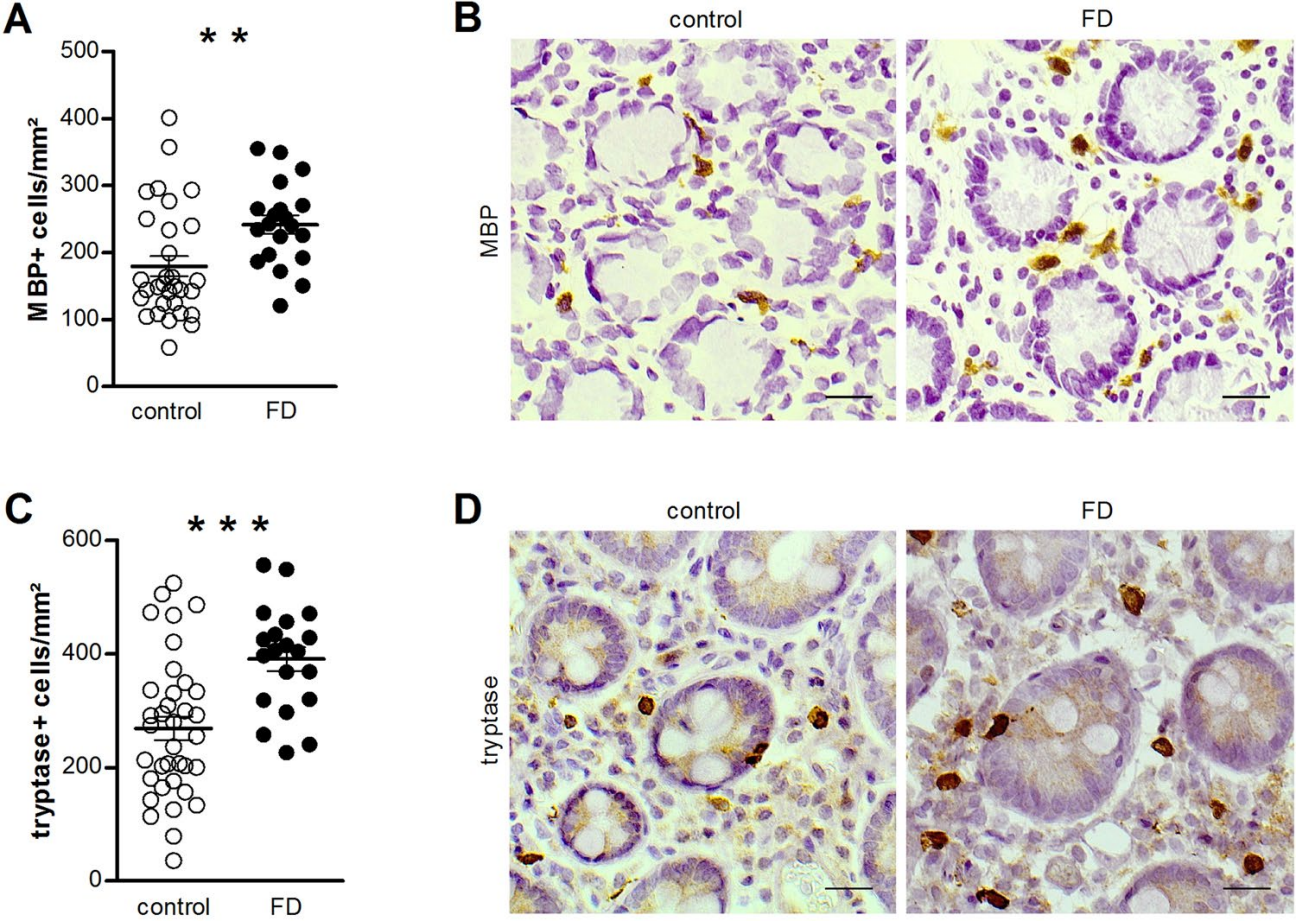
Evaluation of low-grade inflammation. Duodenal biopsy samples from healthy volunteers (control) and patients with FD were stained for identification of eosinophils using anti-human MBP (**A**,**B**) and mast cells using anti-human tryptase (**C**,**D**) antibodies. Cells positive for MBP (n = 31 for controls and n = 20 for patients with FD) (**A**) and tryptase (n = 36 for controls and n = 20 for patients with FD) (**C**) were counted per mm² in at least seven non-overlapping fields per subject. Data are mean ± SEM. Representative images of MBP (**B**) and tryptase (**D**) immunohistochemistry in mucosal biopsy specimens obtained from a control (left) and a patient with FD (right). Scale bar: 20 µm. **p < 0.01, ***p < 0.001. FD, functional dyspepsia; MBP, major basic protein.

The infiltration of eosinophils and mast cells in the lamina propria of duodenal biopsy samples was assessed using immunohistochemistry to evaluate the presence of low-grade inflammation. The results show an increased number of both eosinophils (179.6 ± 15.0 vs. 241.5 ± 14.0 MBP + cells/mm², p = 0.007) (Fig. 1A,B) and mast cells (268.7 ± 20.7 vs. 390.6 ± 20.7 tryptase + cells/mm², p = 0.0003) (Fig. 1C,D) in patients with FD compared with control samples.

### Ultrastructural changes in degranulation of eosinophils and mast cells in functional dyspepsia

Only biopsy specimens with optimal orientation (entire epithelial cells along the apical-basal axis containing lamina propria) and good fixation (absence of mitochondrial damage and intact nuclear membrane structure) were used. Samples from 20 FD patients and 32 controls were optimal for ultrastructural evaluation.

**Figure 2:**
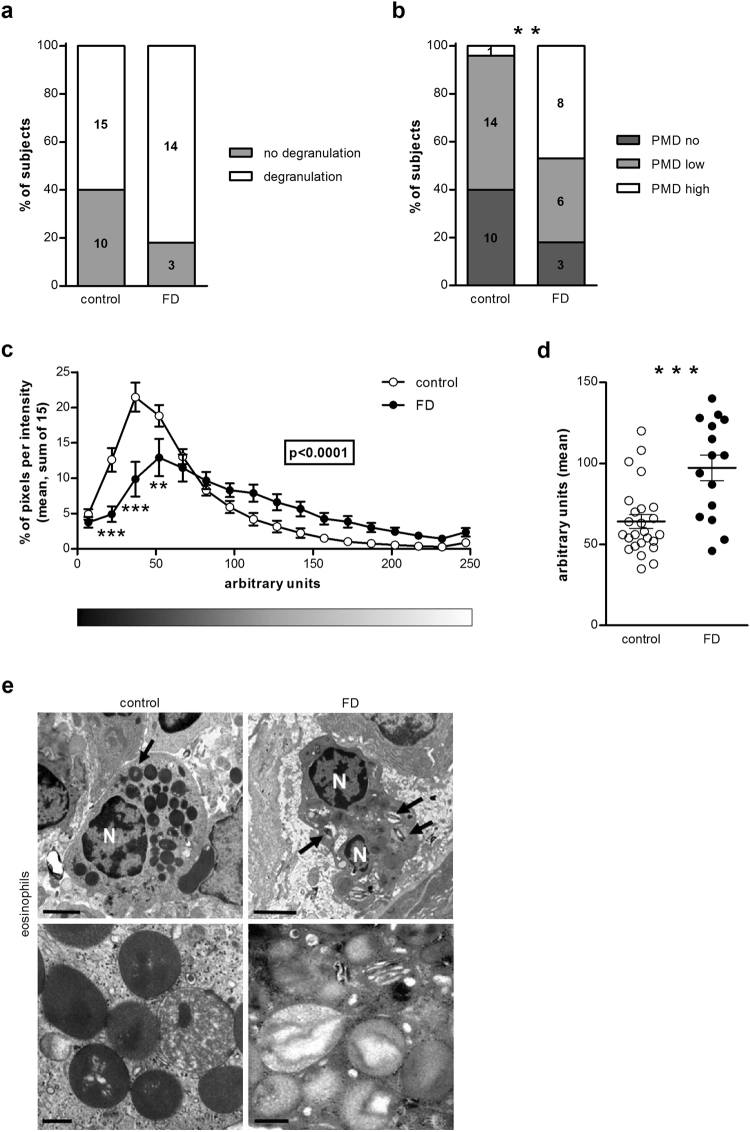
Ultrastructural evaluation of mucosal eosinophils. Duodenal biopsy samples from healthy volunteers (control) and patients with FD were used to evaluate degranulation of eosinophils using transmission electron microscopy. Visual analysis of the presence (n = 25 for controls and n = 17 for patients with FD) (**A**) and degree (n = 15 for controls and n = 14 for patients with FD) (**B**) of degranulation in eosinophils. Quantitative analysis of eosinophilic granular density evaluated as the pixel intensity distribution (**C**) and the mean intensity per granule (**D**) (n = 25 for controls and n = 15 for patients with FD). (**E**) Representative transmission electron micrographs of eosinophils in mucosal biopsy specimens obtained from a control (left) and a patient with FD (right). Lower panels show representative cytoplasmic granules from each group. Note higher loss of granular content in granules from FD group (arrows). Bars indicate magnification (top panels: 2 µm; low panels: 0.5 µm) **p < 0.01, ***p < 0.001. FD, functional dyspepsia; N, nuclei; PMD, piecemeal degranulation.

**Figure 3:**
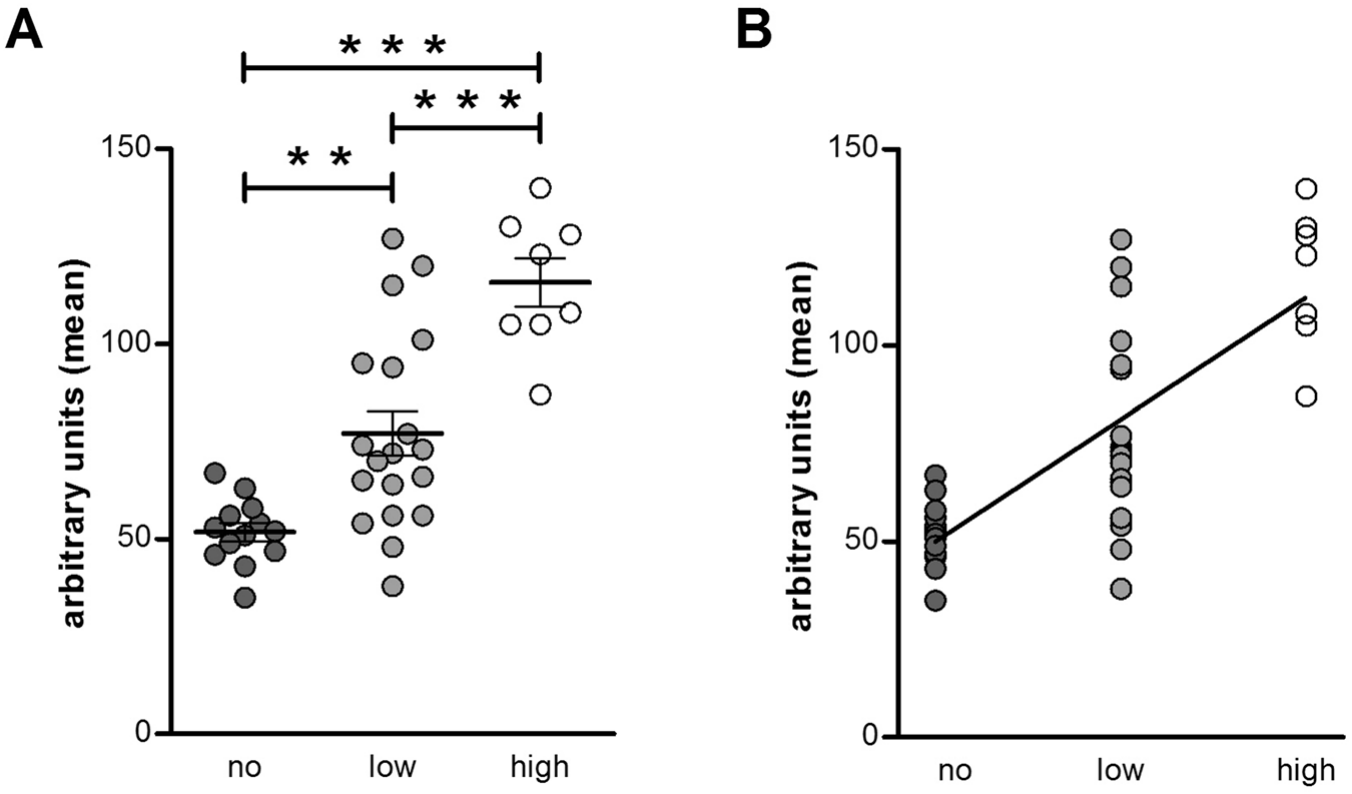
Comparison of visual and quantitative analysis of eosinophilic degranulation. (**A**) The mean intensity per granule was compared between the three groups: no (n = 13), low (n = 19) and high (n = 8) degranulation. (**B**) Correlation between the mean intensity per granule and the degree of eosinophilic degranulation: no (n = 13), low (n = 19) and high (n = 8).

Eosinophils were identified in 78% of control samples and 85% of FD samples. The type of degranulation present in the eosinophils was piecemeal degranulation (PMD), with decreased granular density and core material. No difference in the presence of degranulation of eosinophils was observed between patients and controls (Fig. 2A,E) (p = 0.18). The level of degranulation was, however, more pronounced in the FD group when no, low and high degranulation were compared (Fig. 2B,E) (p = 0.004). Moreover, the quantification of granular density showed that FD patients had less intact granules, illustrated by a shift to the right in the pixel intensity profile (Fig. 2C) (p < 0.0001) and a higher mean intensity per granule (64.2 ± 4.3 vs. 97.3 ± 7.9 arbitrary units, p = 0.0003) (Fig. 2D) compared to control subjects. There was a significant difference in the mean intensity per granule (quantitative analysis) between the three groups ‘no’, ‘low’ and ‘high’ degranulation (visual analysis) (p < 0.0001) (Fig. 3A). The eosinophilic granules of subjects in the ‘no degranulation’ group (51.9 ± 2.3 arbitrary units) displayed a lower intensity per granule than the granules of subjects in the ‘low degranulation’ (77.1 ± 5.7 arbitrary units) (p < 0.01) and ‘high degranulation’ (115.8 ± 6.1 arbitrary units) (p < 0.001) group. The granular density of eosinophils was also lower in the ‘high degranulation’ group compared to the ‘low degranulation’ group (p < 0.001). Moreover, there was a significant correlation between the mean intensity per granule and the degree of degranulation (Fig. 3B) (ρ = 0.76, p < 0.0001). This indicates that the results of the visual and the quantitative analysis are comparable and, therefore, that both methods can be used to evaluate eosinophil activation.

**Figure 4:**
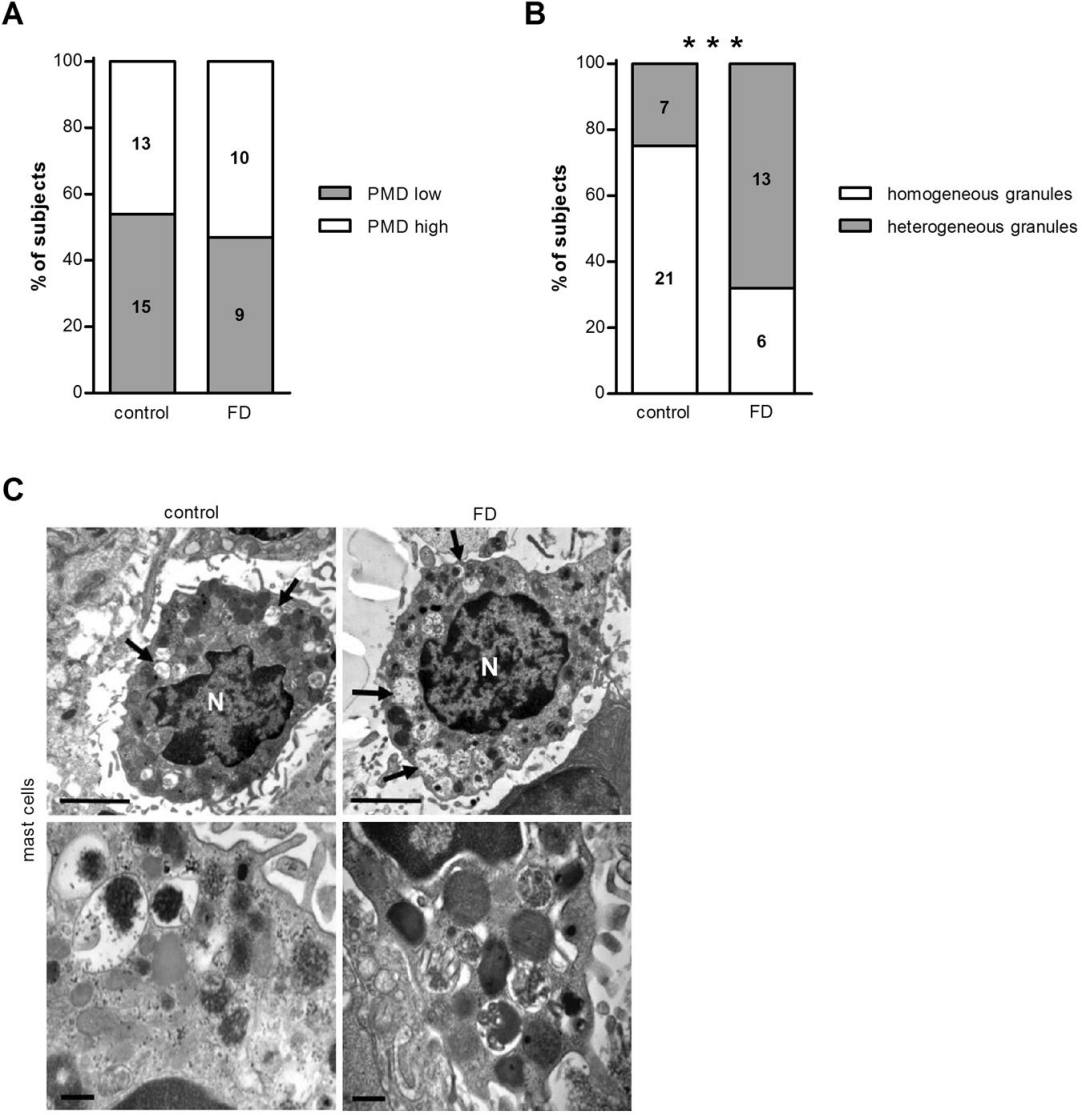
Ultrastructural evaluation of mucosal mast cells. Duodenal biopsy samples from healthy volunteers (control) and patients with FD were used to evaluate degranulation of mast cells using transmission electron microscopy. Degree of degranulation (**A**) and complexity of the granule content (**B**) in mast cells (n = 28 for controls and n = 19 for patients with FD). (**C**) Representative transmission electron micrographs of mast cells in mucosal biopsy specimens obtained from a control (left) and a patient with FD (right). Lower panels show representative cytoplasmic granules from each group. Degranulation is present in cells from both groups (arrows). Bars indicate magnification (top panels: 2 µm; low panels: 0.2 µm). **p < 0.01. FD, functional dyspepsia; N, nuclei; PMD, piecemeal degranulation.

Mucosal mast cells were observed in 28 control samples (88%) and 19 FD samples (95%) and PMD was found in both controls and patients. No difference was observed in the presence of low or high PMD (Fig. 4A,C) (p = 0.77). However, the complexity of the granule content was different in FD patients compared with controls, with a more heterogeneous profile in the FD group (Fig. 4B,C) (p = 0.006), suggesting different granular content of mast cells between groups.

### No association of duodenal epithelial integrity with eosinophils and mast cells

As we previously demonstrated increased duodenal permeability in FD^8^, we wanted to evaluate the relationship between epithelial barrier function and inflammatory activity in these patients. Biopsy specimens from FD patients showed a TEER of 18.0 ± 0.9 Ω.cm² and a paracellular passage of 47.4 ± 3.5 pmol. The number of eosinophils did not correlate with TEER (*ρ* = −0.14, p = 0.61, n = 16) and passage (*ρ* = 0.05, p = 0.86, n = 15). In addition, we did not find a correlation between the degree of eosinophilic degranulation (mean intensity per granule, arbitrary units) and epithelial integrity (TEER: *ρ* = 0.21, p = 0.48, n = 13; FITC-dx4 passage: *ρ* = −0.52, p = 0.08, n = 12). In FD patients, the number of mast cells was not associated with TEER (*ρ* = −0.19, p = 0.48, n = 16), but a negative correlation was detected with passage of FITC-dx4 (*ρ* = −0.55, p = 0.03, n = 15) which did not remain significant after multiple testing correction. There was no difference in TEER (18.0(15.2–20.3) vs. 16.6(15.6–19.2) Ω.cm², p = 0.72) and paracellular passage (38.6(35.6–53.4) vs. 55.8(44.6–63.5) pmol) between FD patients with a homogeneous granular content (n = 6) and those with a heterogeneous granular content (n = 11) of mast cells.

## Discussion

A steadily increasing amount of reports^4^ provide support to the relevance of low-grade inflammation in the duodenal mucosa in at least a subgroup of patients with FD, mainly based on the histological detection of higher numbers of immune cells in the lamina propria. In order to further prove mucosal immune cell involvement, we designed an observational study to assess activation of eosinophils and mast cells in this disorder. Our study describes, for the first time, mucosal immune activation by identifying marked eosinophil degranulation and a more heterogeneous granule content of mast cells in patients with FD compared with healthy controls. We also investigated the relationship between impaired duodenal integrity and inflammatory activity in FD patients, but found no evidence that these pathophysiological mechanisms are associated with each other.

A number of studies have shown the presence of low-grade inflammation in the duodenal mucosa of patients with FD, and hypothesized contribution of an inflammatory mechanism to the pathogenesis of dyspeptic symptoms. The initial trigger for this hypothesis was the observation that a subset of patients develops the disorder after an acute gastroenteritis^14,15^. Based on those findings, various studies have focused on characterizing immune cells in the gastrointestinal tract of patients with post-infectious (PI) FD. A previous study from our group has shown that PI-FD is associated with focal T-cell aggregates, decreased CD4^+^ cells and increased numbers of macrophages in the duodenal mucosa compared to unspecified-onset (U) FD^16^. Futagami and colleagues have also reported increased numbers of activated macrophages and eosinophils in the duodenal mucosa of PI-FD patients compared to controls and increased numbers of macrophages compared to U-FD and controls^17^. Other studies pointed towards immune activation in FD patients as a group, and not only in patients with PI-FD. A population-based case-control study detected increased eosinophil counts in the duodenal mucosa of subjects with FD^3,18^. In line with previous studies, we recently demonstrated increased infiltration of mast cells and eosinophils in the duodenal mucosa of FD patients^8^, which was confirmed in the present study.

The biological function of eosinophils and mast cells mainly relies on mediator release, with most of them active in a concentration-dependent fashion. Thus, the effects of these immune cells are not only dependent on cell density, but also on the extent of degranulation. Previous studies investigating degranulation mechanisms of eosinophils have revealed three basic patterns, which have been termed exocytosis, PMD and cytolysis. During exocytosis, granules release their entire content after fusion of the granule with the plasma membrane. It also involves intergranule fusions before extracellular release. In case of PMD, granules appear empty or partially empty as they undergo progressive emptying of their contents by secretory vesicles, which transport mediators from granules to the cell surface. Lastly, cytolysis is characterized by rupture of the cell membrane and the presence of free granules in the extracellular space^19^. In mast cells, two ultrastructural patterns of degranulation have been described: PMD and anaphylactic degranulation (which can be compared to exocytosis in eosinophils)^20^. The ultrastructural form of secretion of both eosinophils and mast cells in this study was PMD. This was illustrated by variable losses of dense content from cytoplasmic granules, without signs of intergranular or granule-to-cell membrane fusion.

In order to identify the degree of activation of eosinophils and mast cells in FD, we designed an observational study to assess degranulation of these cells. Talley *et al*.^3^ previously evaluated degranulation of duodenal eosinophils in patients with FD and healthy controls using immunohistochemistry, and they found a tendency towards more eosinophil degranulation in the FD group. A more recent study, also using immunohistochemistry, confirmed these results as they reported that FD patients are characterized by increased infiltration of degranulated eosinophils in the duodenal mucosa compared to healthy controls^5^. We, however, chose to use an exclusively morphological study design, based on TEM analysis, to observe the specific granules and to identify the structural changes when they are released. With this technique, it is possible to establish whether the cell is activated and to determine the type of degranulation. One study already investigated duodenal eosinophils using electron microscopy in children with dyspepsia and showed that a significant proportion of patients displayed activated eosinophils^7^. But, since ethical considerations precluded tissue sampling from healthy children, this study lacked a control group. In the present study, using both visual and quantitative analysis, we demonstrated a higher degree of eosinophil degranulation in FD patients than controls. As eosinophils release a diversity of mediators upon activation – such as cationic proteins, cytokines, chemokines and growth factors – increased activation could result in wide-ranging effects^21^.

The assessment of the potential contribution of eosinophil mediators to intestinal dysfunction has not been addressed in the present study, due to the limitation in the number of biopsies collected. In our hands, eosinophil peroxidase (EPO) activity quantification in animal models with low-grade inflammation requires an amount of mucosal tissue of at least 50 mg, in other words, the equivalent to 5–8 duodenal biopsies, making this determination not useful in our experimental setting. To our knowledge, no experimental protocol has been described to specifically process human endoscopic biopsies for eosinophil-derived neurotoxin (EDN), eosinophil cationic protein (ECP) or EPO enzymatic activity determination. Additional studies should be designed in order to identify the type of mediators released by activated eosinophils and mast cells, as they offer a potential interest as therapeutic targets. However, we evaluated whether the mRNA (Supplementary Methods) of specific mediators of the eosinophil (MBP, ECP) were differentially expressed in FD respect to healthy controls, and, contrary to our expectations, none of the molecules assayed revealed statistical differences between groups (Supplementary Figure S1).

In fact, the release of certain mediators, such as MBP and/or tryptase, could be responsible for the identified epithelial barrier dysfunction in FD patients^8^ as previously demonstrated *in vitro*^9,22^. Noteworthy, the lack of association in our study between the number of eosinophils and their degree of degranulation with duodenal integrity points at cellular mediator’s nature as the next unknown factor to characterize in future studies. Eosinophils store and secrete a broad variety of molecules upon stimulation, including cytokines, growth factors proteases and neuro-hormones that can contribute to visceral hypersensitivity as shown *in vitro* and pre-clinical studies in animal models^23,24^. Although not addressed, they could also store and secrete corticotropin-releasing hormone, as has been recently identified in jejunal eosinophils in irritable bowel syndrome, also in association with clinical severity^25^.

In order to investigate whether an increased number of degranulated mast cells play a role in the pathogenesis of FD, an earlier study performed immunostaining of these cells. They did not find a difference in the number of degranulating mast cells in FD patients with respect to controls^5^. Using TEM, we did not detect a difference in the degranulation profile of mast cells between both groups. However, mast cells did clearly show a more heterogeneous granular content in the FD group. While healthy controls displayed particle-containing granules, patients with FD also showed scroll-containing granules which consist of regularly arranged lamellae that form parallel, straight, curved or multilayered figures^20^. The differential granule pattern might reflect a distinct protease composition as it has been shown that the scroll pattern is typical of tryptase-positive, chymase-negative mast cells while more regularly-shaped electron-dense granules indicates the presence of both chymase and tryptase^26^. It can be hypothesized that the granules of patients with FD possess a more deleterious content or that they support the recruitment of other inflammatory cells since it has been reported that tryptase increases eosinophil and neutrophil recruitment^27^, whereas chymase stimulates neutrophil and monocyte accumulation^28^. It is important to notice that the different granule content between healthy subjects and patients might also reflect the presence of a less mature mast cell phenotype in certain specimens. Although it has been shown that mast cell activation can play a role in impaired epithelial barrier function especially in conditions of stress^29^, in the present study, we found no association between the number of mast cells and the complexity of their granular content with impairment of barrier function. Despite the proved role of mast cells and eosinophil mediators in promoting barrier dysfunction, morphological cellular alterations may not represent subsequent epithelial abnormalities. Again, these support the need of designing further research strategies aimed at identifying the type of mediators released by both eosinophils and mast cells in FD patients.

The intent of this study was to perform an initial evaluation of duodenal mucosal eosinophils and mast cells with regard to their activation state to help indicate whether these cells are potentially implicated in the pathophysiology of FD. In keeping with the concept of a duodenal inflammatory component in FD, we demonstrated ultrastructural changes in degranulation of eosinophils and mast cells. Although we did not find an association between activation of these cells and impaired integrity, our results suggest that eosinophil and mast cell activation may play a role in the pathophysiology of FD. The mechanisms by which they influence their environment in FD remain elusive, but the ultrastructural changes outlined in this study can provide a basis for further investigation. Although we believe these results are of major interest, further studies with a higher number of subjects will help to confirm or refute our findings. Ultimately, the clinical relevance of eosinophil and mast cell activation in dyspeptic symptom generation will need to be determined in clinical trials of therapies directed at these immune cells.

## Electronic supplementary material

Supplementary information
