# Supplementary material for: Activation of Eosinophils and Mast Cells in Functional Dyspepsia: an Ultrastructural Evaluation
Source: Sci Rep. 2018 Mar 29;8:5383. doi: 10.1038/s41598-018-23620-y (PMC5876347; doi:10.1038/s41598-018-23620-y)
Supplement: Supplementary file 1 — Supplementary information [file 41598_2018_23620_MOESM1_ESM.docx]

**ACTIVATION OF EOSINOPHILS AND MAST CELLS IN FUNCTIONAL DYSPEPSIA: AN ULTRASTRUCTURAL EVALUATION**

Hanne Vanheel ^1^, Maria Vicario ^2, 3^ *, Werend Boesmans ^1, 4^, Tim Vanuytsel ^1^, Eloisa Salvo-Romero ^2^, Jan Tack ^1^, Ricard Farré ^1, 3^

^1^ Translational Research Center for Gastrointestinal Disorders (TARGID), KU Leuven, Leuven, Belgium; ^2^ Digestive Diseases Research Unit, Institut de Recerca Vall d’Hebron, Department of Gastroenterology, Hospital Universitari Vall d’Hebron, Universitat Autònoma de Barcelona, Barcelona, Spain; ^3^ Centro de Investigación Biomédica en Red de Enfermedades Hepáticas y Digestivas (CIBERehd); ^4^ Department of Pathology, GROW-School for Oncology and Developmental Biology, Maastricht University Medical Center, The Netherlands

* joint first author

**SUPPLEMENTARY METHODS:**

Real-time RT-PCR

Duodenal mRNA was isolated using the RNAeasy Mini Kit (Qiagen), following the manufacturer’s instructions. cDNA synthesis was performed using 1 μg of total RNA with the High Capacity Reverse Transcription Reagents Kit (Thermo Fisher Scientific, Spain), following manufacturer instructions. Transcriptional analysis of MBP and ECP was assessed by qPCR using validated TaqMan Gene Expression Assays (Thermo Fisher Scientific) on an ABI PRISM® 7500 FAST Sequence Detection System (Applied Biosystems). Human cyclophilin A (PPIA) gene was used as the endogenous control for data normalization. Fold change was calculated with respect to the healthy control group using the 2−ΔΔCt method as previously described.^8^

**SUPPLEMENTARY FIGURE:**


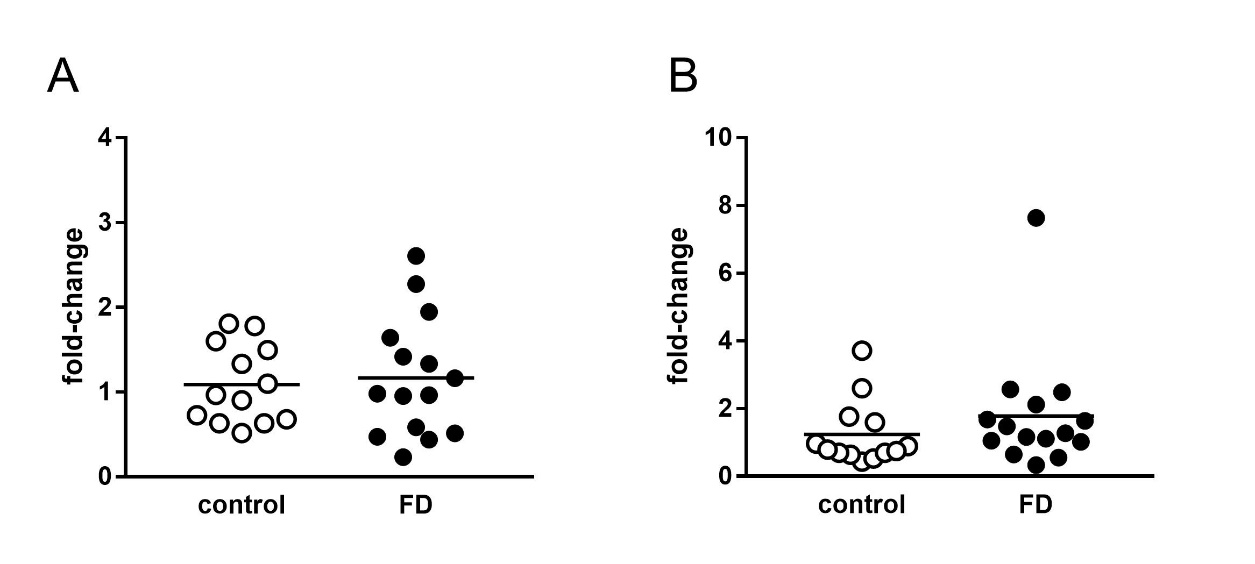


**Figure S1**. Quantification of MBP (A) and ECP (B) mRNA in duodenal biopsies of control and FD patients. Data were normalised to the housekeeping gene, cyclophilin A and expressed as fold-change respect to the average in the control group. No statistical differences were found for MBP (p=0.73) and ECP (p=0.21). MBP, major basic protein; ECP, eosinophilic cationic protein; FD, functional dyspepsia.
